# The Potential Use of Sialic Acid From Edible Bird’s Nest to Attenuate Mitochondrial Dysfunction by *In Vitro* Study

**DOI:** 10.3389/fphar.2021.633303

**Published:** 2021-04-12

**Authors:** Aswir Abd Rashed, Hafandi Ahmad, Siti Khadijah Abdul Khalid, Devi-Nair Gunasegavan Rathi

**Affiliations:** ^1^ Nutrition, Metabolism and Cardiovascular Research Centre, Institute for Medical Research, National Institutes of Health, Ministry of Health, Shah Alam, Malaysia; ^2^ Departments of Veterinary Preclinical Sciences, Faculty of Veterinary Medicine, Universiti Putra Malaysia, Serdang, Malaysia

**Keywords:** edible bird’s nest, sialic acid, cell lines, in vitro, mitochondrial dysfunction, SH-SY5Y

## Abstract

Edible bird’s nest (EBN) is one of the expensive functional foods in herbal medicine. One of the major glyconutrients in EBN is sialic acid, which has a beneficial effect on neurological and intellectual capability in mammals. The aims of this research were to study the effects of sialic acid from EBN on cell viability and to determine its effect on mitochondria membrane potential (MtMP) in Caco-2, SK-N-MC, SH-SY5Y, and PC-12 cell lines. Fourteen samples of raw EBN were collected from four different states in Malaysia. The confluency of the epithelial monolayers measurement of the tight junction for all the cell lines was determined using transepithelial electrical resistance (TEER), and the sialic acid uptake study in cell lines was determined by using ultra-high performance liquid chromatography (UHPLC). The MTT assay was conducted for cell viability study. The MtMP in cell lines was determined using the Mito Probe JC-1 Assay by flow cytometer analysis. We have recorded a statistically significant difference between the uptake of sialic acid from EBN and the standard solution. A higher amount of sialic acid was absorbed by the cells from extract of EBN compared to the standard solution. The amounts of sialic acid uptake in Caco-2, SK-N-MC, SH-SY5Y, and PC-12 cell lines were (0.019 ± 0.001), (0.034 ± 0.006), (0.021 ± 0.002), and (0.025 ± 0.000) µmol/L, respectively. The MTT results indicated that the concentration of sialic acid increased the cell viability and showed no cytotoxicity effects on cell lines when they were exposed to the sialic acid extract and sialic acid standard at all the tested concentrations. The number of active mitochondria was found to be significantly higher in SH-SY5Y cell lines with a 195% increase when treated with sialic acid from EBN. Although many researchers around the globe use SH-SY5Y and SK-N-MC for Alzheimer’s disease (AD) study, based on our finding, SH-SY5Y was found to be the most suitable cell line for AD study by *in vitro* works where it has a known relationship with mitochondrial dysfunction.

## Introduction

Human body is composed of many vital organs, where one of the largest and most complex organs is the brain (Jawabri and Sharma, 2020). Under normal circumstances, brain aging among healthy adults is known to undergo various changes, in terms of structural, chemical, functional, and neuronal alterations. Several of these changes are indicated *via* decrease in brain size, as a whole, as well as declining neurotransmitter systems (Marsman et al., 2013). Despite the fact that all healthy adults are subjected to these changes, it is important to highlight that age-related neurodegenerative disorders are not inclusive as a part of regular healthy aging conditions. These are mainly referred to Alzheimer’s disease (AD) and other forms of dementia (Dobrowolski, 2014).

Alzheimer’s disease or a related form of dementia is estimated to affect approximately 44 million people worldwide (Duthey, 2013). To date, elucidation to demonstrate the mechanism involved in AD pathogenesis is yet to be reported. Amyloid cascade reaction is the most widely recognized action mechanism among the numerous hypotheses that are proposed and available. This reaction puts forward the role of neurotoxic β-amyloid proteins that are deposited within the brain. The presence of these proteins instigates pathological changes that include amyloid plaques aggregation and intracellular neurofibrillary tangles (Murphy et al, 2010). Apart from the explained hypothesis above, there are also evident studies that suggest interrelatedness of mitochondrial damage and AD (Wang et al., 2020). The idea behind this is that the presence of healthy mitochondria is vital for neuron-based activity and also as a protection mechanism to minimize possible oxidative damage (Wang et al., 2020). As such, damaged mitochondria are believed to interfere with these essential roles.

Mitochondria are essential and of high importance for several roles, with their main function channeled toward energy production. The synthesis of high energy molecules in the form of adenosine-5′-triphosphate (ATP) is associated with the presence of mitochondria. The mechanism involved here includes conversion of metabolites energy to reduced nicotinamide adenine dinucleotide (NADH) followed by electron transfer from NADH while protons are also pumped across inner membrane to intermembrane space. This process creates transmembrane potential that utilizes ATP synthase for reflow of protons across inner membrane, and finally this energy is the driving factor of adenosine diphosphate (ADP) phosphorylation to ATP (Nicholls, 2010; Rich and Maréchal, 2010; Divakaruni and Brand, 2011; Bonora et al., 2012). Three factors were proposed as the possible reason that could lead to mitochondrial dysfunction, which include inability to provide required substrates, insufficient mitochondria, and failure of electron transport and ATP synthesis machinery (Nicolson, 2014).

Sialic acid presence is deemed essential for brain development and participates within ganglioside with specific role to enhance learning capability as well as memory (Tram et al., 1997; Wainscot, 2004). Cognitive ability in mammals is related to the variations observed with brain sialic acid concentration. In young mammals, concentrations of ganglioside- and protein-bound sialic acid were enhanced upon exogenous supplementation (Oliveros et al., 2018). Moreover, the exogenous sialic acid is localized to the synapse and the movement of positive neurotransmitters, transmitter release, and altering existing synaptic morphology are influenced by the sialic acid supplementation (Morgan and Winick, 1979). Dietary sialic acid has a role in brain development. Previous study done by Sprenger and Duncan has shown a significant rise in the sialic acid concentration in brain gangliosides and glycoprotein *via* oral administration of sialic acid during an initial postnatal period in the rodents (Sprenger and Duncan, 2012). Another study found that a decline in exogenous sialic acid concentration in brain leads to irreversibly decreased cognitive function but supplementary sialic acid will improve the learning process (Tram et al., 1997). Thus, nutritional interference research is important to evaluate the benefits in digestion and absorption system associated with neurodevelopment function. This will allow detailed analyses of cognitive function and behavior at numerous stages of development and show the association between dietary sialic acid supplementation and cognitive function development (Wang, 2012).

The sialic acid can be found in edible bird’s nest (EBN) and it is one of the eight glyconutrients which has helped to increase cell tissue repair (Kong et al., 1987), promotes cell division and cell proliferation (Aswir and Wan Nazaimon, 2010). EBN has also been reported to be effective in the treatment of neurodegenerative disorders located in the hippocampal and cortical neurons in the brain such as AD and Parkinson’s disease (PD) (Yew et al., 2014; Zhiping et al., 2015), and was able to improve the physiological human health (Guo et al., 2006; Matsukawa et al., 2011). However, extensive research is required to verify the effective levels and safety issues of EBN before it can be marketed more progressively worldwide and consumed by human. This is important because EBN is considered very precious, and its high consumption might not necessarily benefit the body.

In view that there is restricted access to human brain tissue in neuron-based disorders, most researches were channeled toward application of *in vitro* cell line. One of the most common cell lines used is the neuroblastoma (SH-SY5Y) cells. These cells were applied as a prototype for Aβ cytotoxicity in AD (Xie et al., 2010). Another cell line that could also be used is the human induced pluripotent stem cells (iPS) isolated from familial AD (FAD) patient. The iPS cell line is differentiated cells and is hence suitable to be evaluated for presenilin mutation effects (Penney et al., 2020). In addition to the two cell lines, immortal rat hippocampal (IRH) cell lines obtained from embryonic rat hippocampus were also deemed vital as the hippocampal neurons are accountable for both cognitive and memory ability (Eves et al., 1992). Furthermore, previous study by Gilbert and Ross mentioned that this cell line is more beneficial pertaining to its malignant and lack of cell lineage specificity nature, comparatively to tumor cells (Gilbert and Ross, 2009).

As mentioned above, the progression of β-amyloid may result in loss of memory function; thus, the objective of our study was to focus on the effect of sialic acid on mitochondrial dysfunction by using several types of cell lines.

## Materials and Methods

### EBN and Sialic Acid Extract Source

A total of 14 raw unprocessed EBN samples were collected from four states of Peninsular Malaysia representing each region. The samples were collected during the breeding season of edible nest swiftlet within April to August 2016, manually cleaned, and finely grounded using a grinder. The sialic acid was extracted from raw EBN samples at SIRIM Berhad, Malaysia, using the high performance liquid chromatography (Siti Khadijah et al., 2019).

### Cell Lines and Culture Conditions

Four different types of cell lines were used in this study: the colorectal adenocarcinoma (Caco-2/ATCC cat. no. HTB-37); the neuroepithelioma (SK-N-MC/ATCC cat. no. HTB-10); the neuroblastoma (SH-SY5Y/ATCC cat. no. CRL-2266); and the pheochromocytoma (PC-12/ATCC cat. no. CRL-1721). The entire cell lines were purchased from American Type Culture Collection (ATCC, United States). Each of the cell lines was seeded and maintained in 25 cm^2^ culture flasks (Constar, Cambridge, MA) until use.

The PC-12 and SH-SY5Y cells were grown in Dulbecco’s Modified Eagle’s Medium (DMEM; GIBCO New York, United States) supplemented with 20 and 15% v/v fetal bovine serum (FBS), while SK-N-MC cells were grown in Eagle’s Minimum Essential Medium (EMEM; GIBCO New York, United States) supplemented with 20% v/v fatal bovine serum. The entire medium contained 1% v/v nonessential amino acid (GIBCO New York, United States), 1% antibiotic (penicillin–streptomycin) (GIBCO New York, United States), and 1% v/v l-glutamine (GIBCO New York, United States). Only PC-12 cell was added with 15% horse serum (GIBCO New York, United States). All the cells were maintained in the same conditions, at 37°C in an incubator with 5% carbon dioxide, 95% humidity, and air atmosphere. The medium was replaced every 2–3 days. The cells were maintained until they reached 80% confluency.

### Transepithelial Resistance Values Measurement

The cells will reach the maximal levels of differentiation after several days in incubation. To confirm this process, transepithelial resistance values (TEER) can be monitored using EVOM2™ because fully differentiated polarized cells have tight junctions with a TEER of >200 Ω*cm^2^ (MacCallum et al., 2005). The EVOM2™ measures cell monolayer health and cellular confluence *via* qualitative and quantitative measurement, respectively. Before measurement, STX2 electrodes (World Precision Instrument, New Haven, CT, United States) were equilibrated and sterilized according to the manufacturer’s recommendations. Two hundred microliters of culture medium was added to the upper compartment of the cell culture system. The ohmic resistance of a blank (culture insert without cells) was measured in parallel. The blank value was subtracted from the total resistance of the sample, in order to obtain sample resistance. The final unit area resistance (Ω*cm^2^) was calculated by multiplying the sample resistance by the effective area of the membrane (0.33 cm^2^ for 24-well millicell insert plates).

### Cell Viability

The MTT (3-(4,5-dimethythiazol-2-yl)-2,5-diphenyltetrazolium bromide) assay is commonly used to measure cell viability and proliferation (Ahmad et al., 2006). Adherent cells were dissociated from their substrate by trypsinization or scraping. Then, the cells were centrifuged at 830 × g for 5 min. The supernatants were discarded, and the cell pellet was resuspended in DMEM at a density of 20,000 cells/cm^2^ into a 96-well plate. The cells were then incubated overnight in an incubator with 5% carbon dioxide (CO_2_), 95% air atmosphere, and 95% relative humidity at 37°C. The next day, the medium was changed to medium-free FBS following exposure to (or treatment with) sialic acid at the required concentration (20, 40, 60, 80, and 100 μg/ml), and the cells were incubated for 24 h. MTT reagent at a concentration of 5 mg/ml and a volume of 10 µL was added to each well and incubated for 3–4 h until the purple precipitate was visible. Later, the medium was aspirated from the wells as completely as possible without disturbing the formazan crystals and cells on the plastic surface. Then, 100 µL of dimethyl sulfoxide (DMSO) was added to each well, followed by agitation on a plate shaker for 5 min, and finally, the optical density was read at 570 nm. The number of surviving cells is directly proportional to the level of the formazan product created (Van de Loosdrecht et al., 1994).

The absorbance value for the blanks should be 0.00 ± 0.1 optical density (O.D) units. The absorbance range for untreated cells should typically be between 0.75 and 1.25 O.D. units. Selection of a cell number (i.e., providing values between the range of 0.75 and 1.25) allows for the measurement of both stimulation and inhibition of cell proliferation. If the absorbance values of the experimental samples are higher than the control, this indicates an increase in cell proliferation. Alternatively, if the absorbance rates of the experimental samples are lower than the control, this indicates a reduction in the rate of cell proliferation or a reduction in overall cell viability.

### Sialic Acid Uptake Studies in Caco-2, SK-N-MC, SH-SY5Y, and PC-12 Cell Lines

For the purpose of sialic acid uptake studies, the cells were grown on transwell plates for 21 days. The culture medium was removed before adding 2 ml of uptake buffer (140 mM NaCl, 5 mM KCl, 1 mM NaH_2_PO_4_, 10 mM Mes, 0.5 mM of MgCl_2_, and 1.0 mM CaCl_2_ at pH 6.0) and incubated at 37°C for 2 min. Later, 1 ppm (∼3 μmol/L) of sialic acid extract was added to each well and incubated for 15 min at 37°C. Then, the buffer was aspirated, and cells were rinsed with cold buffer for three times before adding 1 ml of 200 mM NaOH to solubilize the cells and they were left overnight at 4°C. After an overnight incubation at 4°C, the cells were subsequently removed; the levels of sialic acid in buffer and cells were determined by using UHPLC. This procedure follows the method of Derakhshandeh and his colleagues with slight modification (Derakhshandeh et al., 2011).

### Mitochondrial Membrane Potential

This procedure was performed using the Mito Probe JC-1 Assay Kit for flow cytometry (Life Technology, United States). The kit contains 30 µg powdered dye, DMSO, carbonyl cyanide 3-chlorophenylhhydrazone (CCCP), and 10× phosphate-buffered saline (PBS). The cell lines were fixed with 150 µL of sialic acid extract and standards for 24 h prior to staining the cells for flow cytometer measurement. In brief, all reagents were equilibrated at room temperature before beginning the experiment. Two hundred micromolar of JC-1 powder stock solution was prepared by dissolving the vial with 230 µL of the DMSO. Then, 1 × 10^6^ cells/mL of cell lines were collected by scrapping the cells and centrifuged in warm PBS before being incubated at 37°C for 5 min. To provide the positive control, 1 µL of 50 mM of CCCP (50 µM in final concentration) was added to the tube and incubated for 5 min. After the incubation, 10 µL of JC-1 stock solution (2 µM in final concentration) was added to all tubes and incubated for another 30 min at 37°C. Later, all cells were washed using the warm PBS and centrifuged to obtain the cell pellet. Then, the cells were resuspended by adding 500 µL of PBS to each tube. All tubes were analyzed using a flow cytometer machine (BD FACSAria™) with 488 nm excitation using emission for Alexa Fluor® 488 dye and fluorescence microscopy. The JC-1 dye detection was used with bandpass filters centered around 529 nm (green fluorescence) and bandpass filters centered around 595 nm (orange fluorescence). The logarithmic signal amplification was used with the typical green–orange electronic signal compensation near 4% and orange–green signal compensation around 10% (Cossarizza and Salvioli, 2001). The depolarization of mitochondria in cells is indicated by decreasing ratio of fluorescence intensity (red JC aggregates/green JC monomer).

### Statistical Analysis

All data were analyzed using the SPSS software version 16 (IBM Software, Inc., New York, United States). The viability of the cell lines induced with sialic acid was analyzed using the one-way analysis of variance (ANOVA) with post hoc Tukey’s test to show significant difference between groups. *p* < 0.05 was considered statistically significant.

## Results

### Transepithelial Resistance Values Measurement

**FIGURE 1:**
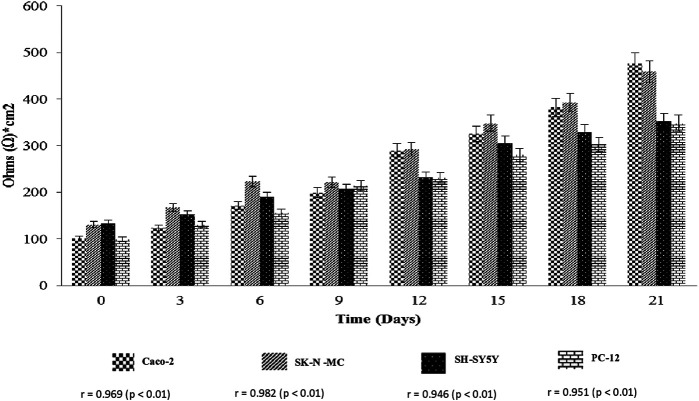
Transepithelial electrical resistance assay (TEER) (Ohms*cm^2^) measurement of cell lines. Values are expressed as mean ± SEM of triplicate experiments.

**FIGURE 2:**
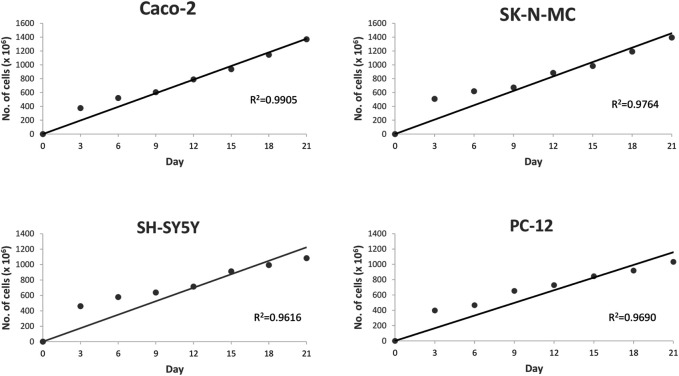
Scattered plot shows the correlation between cell lines and day of culture. Values are given as 1 × 10^6^ (mean ± SEM) for three independent biological determinations.

The determination of cell cultures confluency stage for entire cell lines using EVOM2™ was shown in Figure 1, 2. Caco-2 and SK-N-MC cell lines reached their 50% confluency at day 12 with 289.91 ± 137.89 Ω*cm^2^ and 292.71 ± 80.61 Ω*cm^2^, respectively, while SH-SY5Y and PC-12 cell lines reached their 50% confluency at day 15 with 305.91 ± 80.61 Ω*cm^2^ and 280 ± 127.98 Ω*cm^2^, respectively. The Pearson Rank correlation analysis was performed to determine the relationship between the days of culture and TEER reading. Based on results in Figure 2, a strong positive correlation between culture duration and all cell lines was recorded (*p* < 0.01).

### Cell Viability

Table 1 shows the effect of sialic acid extract and standard at 20, 40, 60, 80, and 100 μg/ml concentrations on cell viability in serum-free medium, which represents the conditions for the sialic acid uptake study. The cell viability results showed that there are no cytotoxicity effects in all neuroblastoma and epithelial cell lines when exposed to sialic acid at the concentration of 60 μg/ml or below. All cell lines showed significant differences in cell viability (*p* < 0.05).

**TABLE 1:** Effects of sialic acid from EBN on cell lines in 1 × 10^6^ cells/ml.

Treatment concentration (µg/ml)	Cell viability (%)							
	Sialic acid extract				Sialic acid standard			
	Caco-2	SK-N-MC	SH-SY5Y	PC-12	Caco-2	SK-N-MC	SH-SY5Y	PC-12
0	8.9 ± 4.48^c^	9.27 ± 0.61^d^	9.3 ± 1.57^d^	9.6 ± 1.76^d^	9.6 ± 1.99^b^	9.0 ± 4.69^d^	9.5 ± 6.22^c^	9.5 ± 2.50^c^
20	79.13 ± 3.01^b^	71.6 ± 4.38^b^	61.63 ± 1.76^b^	52.0 ± 8.58^c^	65.93 ± 3.62^a^	65.57 ± 2.48^b^	54.0 ± 1.82^b^	39.53 ± 4.89^b^
40	77.67 ± 5.52^b^	77.33 ± 3.48^b^	64.57 ± 2.55^b^	63.93 ± 3.62^b^	63.9 ± 5.87^a^	73.03 ± 0.81^b^	54.0 ± 3.01^b^	57.5 ± 6.54^a^
60	90.2 ± 5.46^a^	86.57 ± 4.85^a^	81.93 ± 8.65^a^	72.53 ± 2.67^a^	78.23 ± 3.21^a^	82.27 ± 0.91^a^	65.77 ± 1.94^a^	63.57 ± 4.25^a^
80	74.47 ± 4.77^b^	83.77 ± 4.56^a^	53.3 ± 1.28^c^	63.33 ± 4.95^b^	69.07 ± 9.81^a^	79.43 ± 3.17^a^	51.0 ± 3.76^b^	61.37 ± 7.90^a^
100	70.6 ± 2.65^b^	64.77 ± 5.46^c^	49.17 ± 5.06^c^	62.00 ± 2.91^b^	64.67 ± 12.40^a^	70.77 ± 6.76^b^	43.46 ± 8.51^b^	53.37 ± 7.03^a^

Cells were treated with sialic acid extract and sialic acid standard for 24 h in serum-free medium. The optical density was determined by spectrophotometer at 570 nm. Values are expressed as mean ± SEM of triplicate experiment. ^a, b, c, d^ Means without common letters in their superscript are significantly different (*p* < 0.05).

The percentage of cell viability in epithelial and neuroblastoma cell lines was significantly higher when they were induced with sialic acid extract compared to sialic acid standard. Above the concentration of 60 μg/ml, all cell lines showed negative effect of sialic acid on cell viability.

### Sialic Acid Uptake Studies in Caco-2, SK-N-MC, SH-SY5Y, and PC-12 Cell Lines

Table 2 shows the concentration of sialic acid that has been absorbed by the entire cell lines after selected concentration of sialic acid was added to the cells. For cell monolayer, all cell lines showed significant differences between the extraction and control (*p* < 0.05) and between the extraction and standard extraction (*p* < 0.05), respectively. However, only Caco-2 cell lines showed a significant difference between the control and standard (*p* < 0.05). For basal solution, the mean absolute difference (MD) of sialic acid uptake was significant between the control and the extraction in all cell lines. The MD of sialic acid uptake was also significant between the extraction and the standard. In contrast, only SH-SY5Y cell line has recorded the differences in sialic acid uptake between the control and the standard.

**TABLE 2:** Sialic acid uptake studies by Caco-2, SK-N-MC, SH-SY5Y, and PC-12 cell lines.

Treatment	Sialic acid uptake							
	Basal solution (µmol/L)				Cell monolayer (µmol/L)			
	Caco-2	SK-N-MC	SH-SY5Y	PC-12	Caco-2	SK-N-MC	SH-SY5Y	PC-12
Control	0.003 ± 0.002^b^	0.003 ± 0.003^b^	0.004 ± 0.001^c^	0.003 ± 0.000^b^	0.003 ± 0.001^c^	0.003 ± 0.005^b^	0.003 ± 0.08^b^	0.002 ± 0.000^b^
Extraction	0.020 ± 0.001^a^	0.055 ± 0.006^a^	0.043 ± 0.002^a^	0.043 ± 0.000^a^	0.019 ± 0.001^a^	0.034 ± 0.006^a^	0.021 ± 0.002^a^	0.025 ± 0.000^a^
Standard	0.006 ± 0.002^b^	0.004 ± 0.006^b^	0.027 ± 0.003^b^	0.003 ± 0.000^b^	0.012 ± 0.001^b^	0.004 ± 0.001^b^	0.009 ± 0.003^b^	0.003 ± 0.000^b^

Values are expressed as mean ± SEM in triplicate experiment. ^a, b, c^ Means without common letters in their superscript are significantly different (*p* < 0.05).

### Mitochondrial Membrane Potential

**FIGURE 3:**
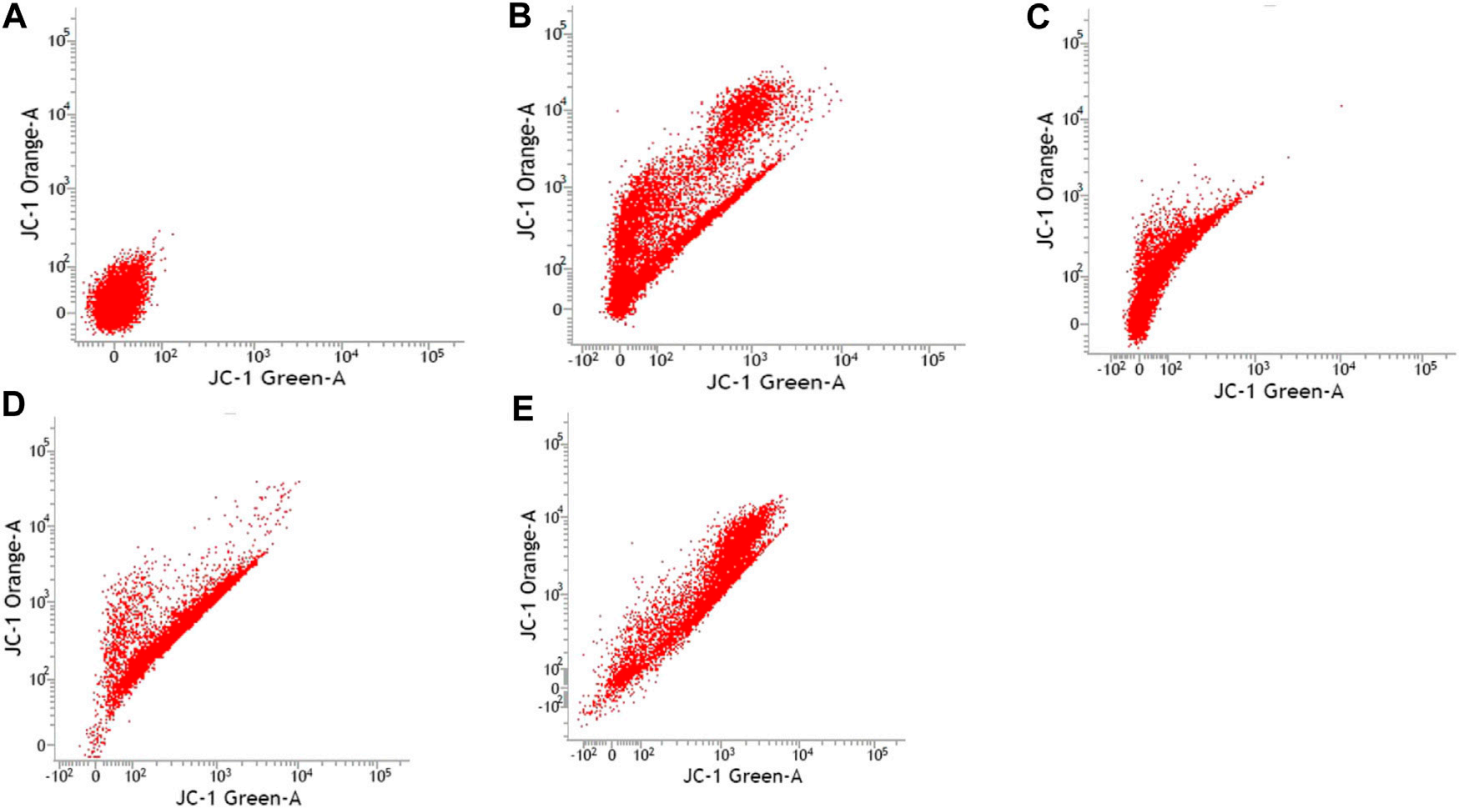
Caco-2 cell lines with or without treatment with sialic acid using JC-1 Mito Probe by BD FACSAria™: **(A)** untreated cell; **(B)** control without CCP; **(C)** control with CCP; **(D)** treatment with standard sialic acid; **(E)** treatment with extraction of sialic acid.

**FIGURE 4:**
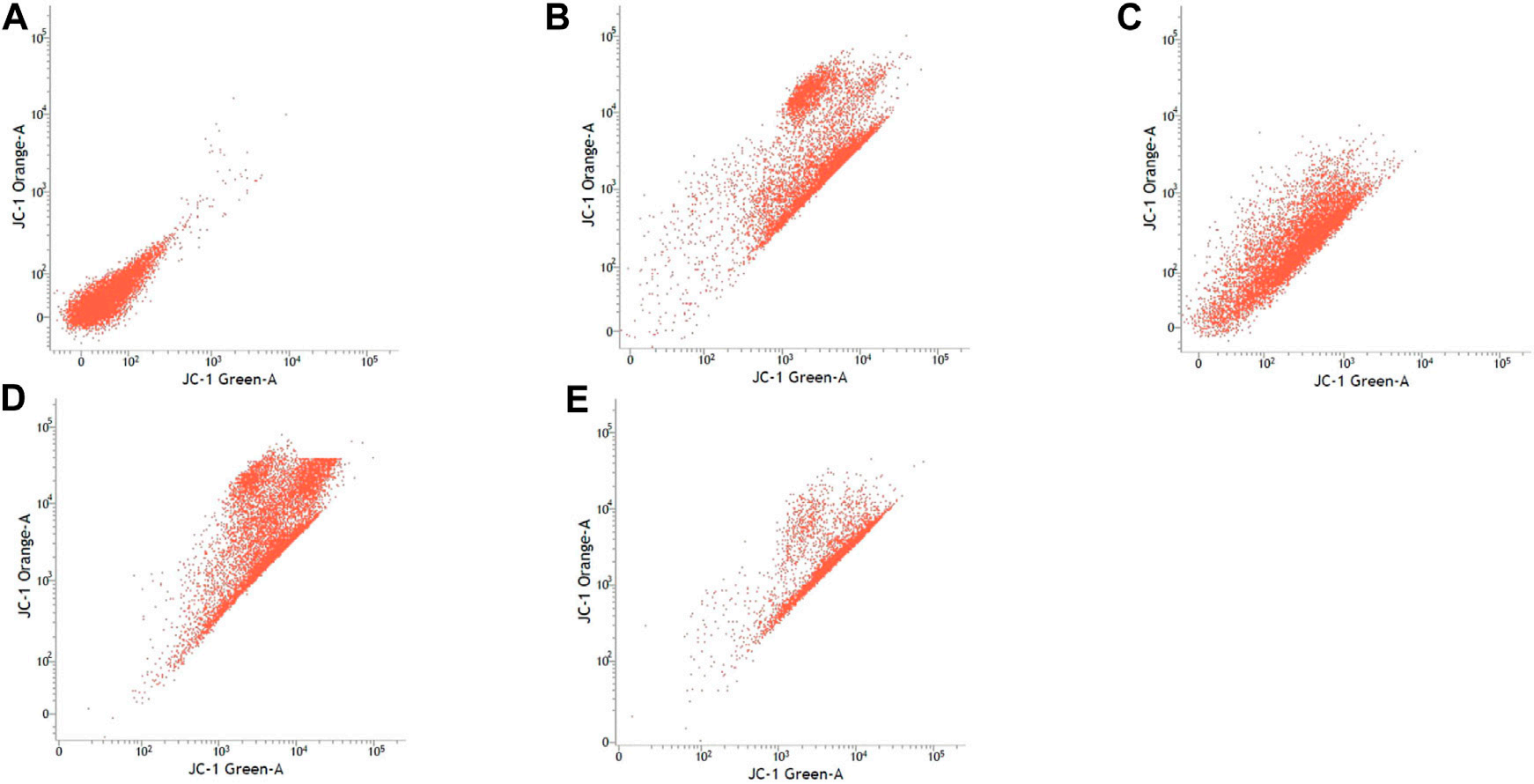
SK-N-MC cell lines with or without treatment with sialic acid using JC-1 Mito Probe by BD FACSAria™: **(A)** untreated cell; **(B)** control without CCP; **(C)** control with CCP; **(D)** treatment with standard sialic acid; **(E)** treatment with extraction of sialic acid.

**FIGURE 5:**
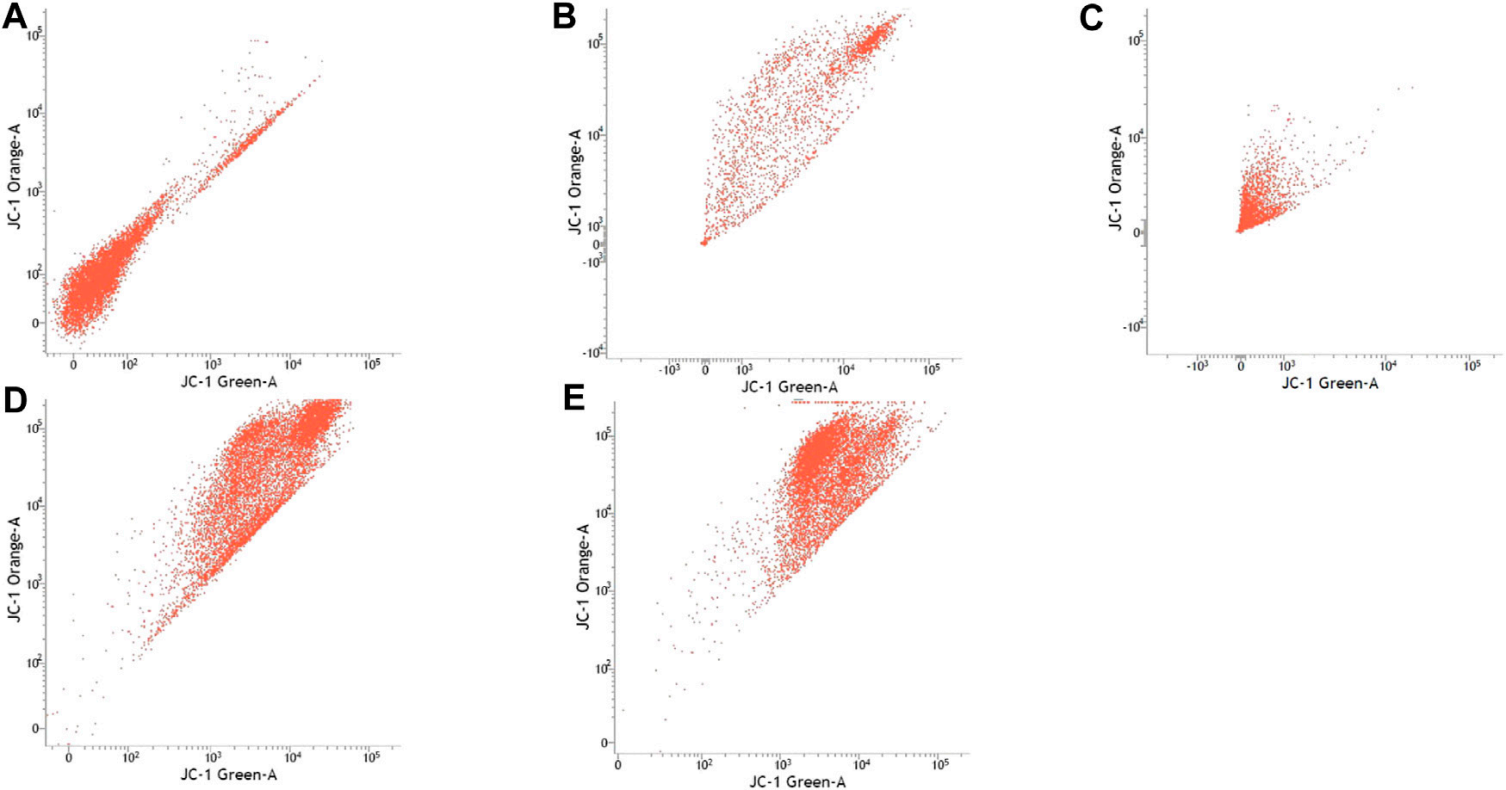
SH-SY5Y cell lines with or without treatment with sialic acid using JC-1 Mito Probe by BD FACSAria™: **(A)** untreated cell; **(B)** control without CCP; **(C)** control with CCP; **(D)** treatment with standard sialic acid; **(E)** treatment with extraction of sialic acid.

**FIGURE 6:**
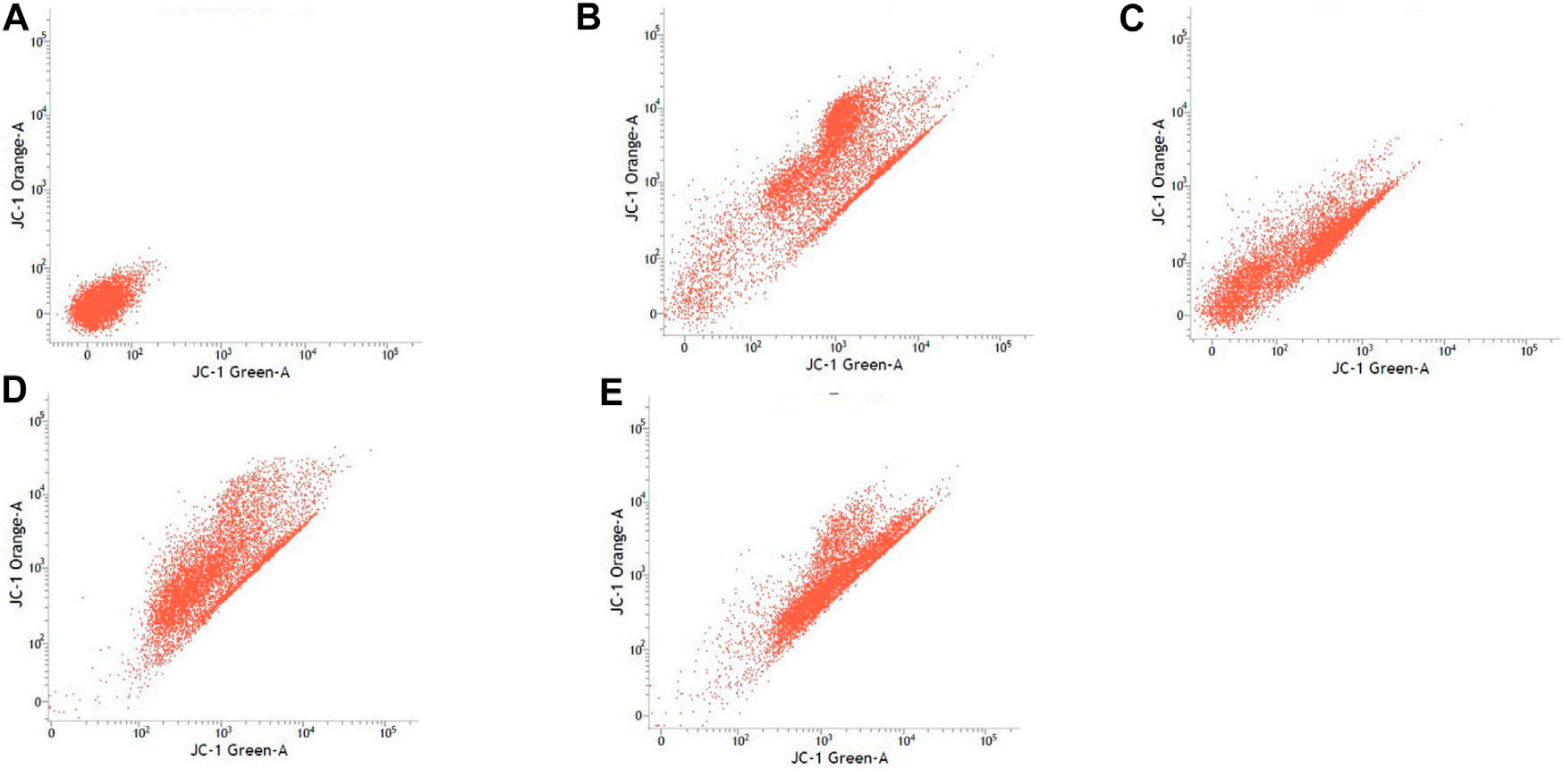
PC-12 cell lines with or without treatment with sialic acid using JC-1 Mito Probe by BD FACSAria™: **(A)** untreated cell; **(B)** control without CCP; **(C)** control with CCP; **(D)** treatment with standard sialic acid; **(E)** treatment with extraction of sialic acid.

Table 3 shows the percentage of active mitochondria once being treated with sialic acid in cell lines using flow cytometry analysis. Figures 3–6 present the excitation peak of entire cell exposed to JC-1 dye using 488 nm wavelength. One-way ANOVA showed that there was a significant difference in numbers of active mitochondria between all groups of treatment (*p* < 0.05). The Tukey post hoc test revealed that the number of active mitochondria in SH-SY5Y is significantly higher when induced with sialic acid compared with control (*p* = 0.000). However, even without treatment with sialic acid, all other cell lines have a higher number of active mitochondria at the start of the experiment.

**TABLE 3:** The numbers of mitochondria in flow cytometer analysis for Caco-2, PC-12, SH-SY5Y, and SK-N-MC cell lines.

Types of cell lines	Numbers of mitochondria (%)		
	Control	Sialic acid standard	Sialic acid extract
Caco-2	96.04 ± 0.03^a^	98.10 ± 0.94^a^	80.40 ± 0.55^b^
PC-12	95.86 ± 1.15^a^	92.98 ± 1.00^a^	91.96 ± 0.65^a^
SH-SY5Y	48.34 ± 1.22^b^	96.39 ± 0.61^a^	90.56 ± 0.85^a^
SK-N-MC	93.76 ± 0.87^a^	93.51 ± 0.70^a^	94.80 ± 0.30^a^

Values are expressed in mean ± SEM for three independent experiments. ^a, b^ Means without common letters in their superscript are significantly different (*p* < 0.05).

## Discussion

In studies that involve cell line usage, it is important to control possible variations especially in terms of standardizing optimal duration after seeding as this ensures generation of differentiated cultures with excellent uniformity. Furthermore, development of tight junction in these cells is highly associated with the culture duration (Srinivasan et al., 2015). As an example, Caco-2 cells are derived from colorectal adenocarcinoma cells and spontaneously undergo differentiation between 0 and 21 days. It is important to also note that the Caco-2 cells are nonidentical to normal duodenal enterocytes (Mahraoui et al., 1994; Sharp et al., 2002; Mariadason et al., 2000). Based on reported literature, fully differentiated polarized Caco-2 cells have tight junctions with a TEER value of >200 Ω*cm^2^, and as such the differentiation process is confirmed by careful monitoring of the cell’s polarization process (Eves et al., 1992). On the other hand, fully differentiated neuroblastoma cells (SH-SY5Y) express various markers of mature neurons. These are inclusive of growth-associated protein (GAP-43), neuronal nuclei (NeuN), synaptophysin (SYN), synaptic vesicle protein II (SV2), neuron specific enolase (NSE), and microtubule associated protein (MAP) (Murillo et al., 2017). At the same time, the fully differentiated cells also indicate that they lack expression of glial markers such as glial fibrillary acidic protein (GFAP) (Liu et al., 2009).

TEER measurement is subject to certain level of variations, especially among distinct groups in different studies. These variations observed are possibly contributed by a number of factors. The identified factors include difference in actual junction tightness, temperature, cells handling technique during measurements, and potential difference in measuring equipment (e.g., chopstick or cup electrodes, impedance measurements). In addition, it should also be highlighted that translating TEER into a functional estimate of tightness is tough. This is due to the fact that composition of the tight junction complexes and the size of the compound of interest are the underlying factors that influence the endothelial monolayer tightness (Srinivasan et al., 2015; Helms et al., 2016). In our study, we found that the human epithelial cell lines (Caco-2) and human brain barrier cell lines (SK-N-MC, SH-SY5Y and PC-12) reached more than 300 Ω*cm^2^ at 21 days. In comparison to previous reported review by Helms and colleagues, our finding is considered higher than the reported standard range of TEER values for blood–brain barrier cell culture, which is around 40–200 Ω*cm^2^ (Helms et al., 2016).

In comparison to *in vivo* studies, *in vitro* cytotoxicity and cell viability assays were shown to be more advantageous in terms of speed, lower cost, and automation potential. Due to these reasons, studies conducted with human cells are deemed more appropriate than *in vivo* research (Chrzanowska et al., 1990). MTT assay is a colorimetric assay that assesses the cell metabolic activity. Before any dietary components are investigated for their effects on iron uptake in Caco-2 cells, it is prudent to evaluate cell viability under incubation conditions and the reduction of tetrazolium salts, as part of the MTT assay, which is now recognized as a safe and accurate test for cell viability (Yew et al., 2014). NADPH and NADH that are synthesized by dehydrogenase enzymes in metabolically active cells are responsible for this conversion (Xu et al., 2015). It was found that the cell viability of the Caco-2, SK-N-MC, PC-12, and SH-SY5Y cell lines were upregulated alongside the steady increase in the concentration of sialic acid in the media. At 1 × 10^6^ cell lines, sialic acid extract and sialic acid standard did not show any cytotoxicity effects on cell viability up to a concentration of 60 μg/ml. However, above 80 μg/ml concentration, there was a reduction in cell viability for all the cell lines. This result is similar to our previous published finding on cell proliferation where it was concluded that stimulation with different concentration of sialic acid from EBN and sialic acid standard into cells will give rise to a dose dependent increase in cell viability (Aswir and Wan Nazaimon, 2011).

The cell viability of Caco-2 and SH-SY5Y cell lines showed remarkable difference when treated with sialic acid extract compared to the standard sialic acid. This discrepancy could be due to the variation in extraction process. Besides, there are many different types of standard sialic acids commercially available and containing a mixture of sialic acids found in humans and animals. The extraction of standard sialic acids also varies from sulfuric acid, phosphoric acid, acetic acid, trifluoroacetic acid (TFA), and HCl. None of the sialic acid on the market is obtained from bird’s nests. Since bird’s nests naturally contain higher bioactive compounds including sialic acid, it may be one of the reasons why sialic acid obtained from bird’s nest has a slightly higher absorption rate than the commercially available sialic acid. In addition, the increase in cell viability with higher concentration might be influenced by the mitogenic properties in sialic acid extract from EBN that promoted the cell growth as manifested by previous studies which showed the enhanced cell division in rabbit corneal keratocytes using EBN (Zainal Abidin et al., 2011; Yew et al., 2014). Although sialic acid standard also showed increase in cell viability, this effect was observed to be slightly lower than the sialic acid extract. This could be due to low activities associated with the varied process and preparing the treatment as previously mentioned (Yew et al., 2014). Moreover, the quantity of absorbance signal created is dependent on numerous parameters including the concentration of MTT reagent, the time of incubation period, the numbers of viable cells, and also their metabolic activities (Riss et al., 2016).

In general, sialic acid rarely exists free in nature and is usually available as component of oligosaccharide chains of mucins, glycoproteins, and glycolipids. In terms of its position, sialic acid usually occupies terminal, nonreducing positions that are highly exposed and functionally essential. These commonly refer to nonreducing positions of oligosaccharide chains of complex carbohydrates on both outer and inner membrane surfaces, mainly to galactose, N-acetylgalactosamine, and other sialic acid moieties. The highest concentration of sialic acid is present in mammalian central nervous system, where majority of it is found in gangliosides (65%), followed by glycoproteins (32%), while the remaining exists as free sialic acid (Brunngraber et al., 1972).

To date, there are limited findings on digestion and mechanisms involved among sialic acid compounds. It was reported that rat intestinal walls are highly permeable to free sialic acids. In addition, it was highlighted that sialidases of bacterial origin could possibly cleave sialic acid residues from milk oligosaccharides in colon; however it was not evident if sialic acid is capable of absorption across colonic mucosa (Wang and Brand-Miller, 2003). Hence, a cell model was established in order to understand and evaluate the mechanisms involved in sialic acid transport across cellular barriers. One relevant cell model is the Caco-2 epithelial cells that plays its role in studying transport from gastrointestinal (GI) lumen into the blood (Wilson et al., 1990), where sialic acid is taken orally. In this study, we determine the concentration of sialic acid that has been absorbed by the cell using UHPLC instead of radiolabeled isotope. A monolayer of Caco-2 cells was applied as the model uptake prototype to demonstrate sialic acid uptake across the GI epithelium. The same model was applied for sialic acid uptake in the brain through a monolayer of SK-N-MC, SH-SY5Y, and PC-12 cell lines.

Based on literature, it was found that the normal range of total sialic acid (TSA) level found within serum or plasma falls in the range of 1.58–2.22 mmol/L. From this, only about 0.5–3.0 μmol/L corresponds to free form of SA (Sillanaukee et al., 1999). Thus, we used 3 μmol/L of sialic acid extract to mimic the normal range of free sialic acid in the human body. Our study has recorded sialic acid uptake by the cell lines. A number of transport mechanisms across cytoplasmic membrane have evolved in response to sialic acid transport in bacteria. Tripartite ATP-independent periplasmic (TRAP), ATP binding cassette (ABC), major facilitator superfamily (MFS), and sodium solute symporter (SSS) transporter families are among the common identified mechanisms (Vimr and Troy, 1985; Allen et al., 2005; Post et al., 2005; Severi et al., 2010; North et al., 2017; Wahlgren et al., 2018). Based on our finding, there is high possibility of the presence of sialic acid transporter in monolayer cell lines that helps in the transportation of sialic acid extract across the membrane to the basal chamber. From the original concentration of 3 μmol/L, we found that high concentration of sialic acid was recorded in neuroblastoma (SK-N-MC, SH-SY5Y, and PC-12) cell lines compared to epithelial (Caco-2) cell lines. Although we did not perform a study on the sialic acid transporter, this finding, however, shed some lights and indirectly gave insights for the reason behind high sialic acid concentration in brain compared to the other parts of the body. The previous study done by Bardor and his colleagues (Bardor et al., 2005) which reported that human neuroblastoma cell lines could be incorporated with sialic acid with efficiency comparable with Caco-2 cells also supports our findings. Thus, this could suggest that the mechanism of sialic acid uptake study can also occur in other human cells but with varying degrees.

Eukaryotic cells contain several types of organelles, which may include nucleus, mitochondria, chloroplasts, the endoplasmic reticulum, the Golgi apparatus, and lysosomes. Each of these organelles performs a specific function critical to the cell's survival. Since cell lines were also obtained from the eukaryotic cells, they also have the same organelles. Mitochondria study gained attention in 1970s, when research was focused on studying energy conservation mechanism as well as ATP synthesis. At the same time, chemiosmotic theory of oxidative phosphorylation was also established, which led to conferment of Nobel Prize in Chemistry awarded to Peter Mitchell in year 1978 (Brand et al., 2013). Any part of the body can be affected by mitochondrial diseases such as the cells of the brain, nerves, muscles, kidneys, heart, liver, eyes, ears, and pancreas. Mitochondrial dysfunction arises upon failure of the mitochondria in ATP synthesis due to certain underlying conditions or diseases. These diseases can lead to secondary mitochondrial dysfunction, including but not limited to Alzheimer’s disease, muscular dystrophy, diabetes, and cancer (Brand et al., 2013).

In this study, the JC-1 was used because it is more specific for measuring changes in mitochondria membrane potential (MtMP) (De Biasi et al., 2015). It is also consistent in response to the depolarization of the mitochondria compared to other cationic dyes such as Rhodamine-123 and 3,3′ dihexyloxacarbocyanine iodide (DiOC6) which are toxic to the mammalian cells (Shapiro, 1994). The carbonyl cyanide 3-chlorophenylhhydrazone (CCCP) was used as a control to confirm that JC-1 dye responses to membrane potential fluctuations and also determines compensation percentage that is necessary in order to quantify 488-excited J-aggregates accurately (Perelman et al., 2012; Sivandzade et al., 2019). Throughout oxidative phosphorylation, essential components that are required in energy storage are the MtMP derived by proton pumps, specifically called Complexes I, III, and IV (Zorova et al., 2018). Based on our findings, Caco-2, PC-12, and SK-N-MC cell lines revealed a high number of active mitochondria compared to SH-SY5Y cell lines in control group (Table 3). The sialic acid extract exhibited less numbers of active mitochondria in Caco-2 cells compared to sialic acid standard in respond to the sialic acid stimulus. The impact developed and was upregulated almost 100% when sialic acid was added to the cells compared to the control.

Previous study showed that the mitochondrial functionality can be restored by natural product such as herbal medicine which helps in preserving the dopaminergic neurons in Parkinson disease (Kim et al., 2004), and in this study the sialic acid caused the depolarization of the mitochondria cells. The percentage of mitochondria was slightly increased when sialic acid standard was added to the media containing Caco-2 cell lines compared with the control. However, the percentage was reduced significantly when sialic acid extract was added to the media. Although these findings were interesting, the root cause of this phenomenon is unknown. Conversely, in the SK-N-MC cell line, the percentage of mitochondria was slightly higher in sialic acid extract (94.80%) compared to sialic acid standard (93.51%). This is because the SK-N-MC is a human brain cell and according to Schauer (Schauer, 1982), neural cell in membrane contains 20 times more sialic acid than other types of membrane and clearly indicated that sialic acid is of utmost importance in neuronal development. The results were similar to the study done by Rosernberg (Rosenberg, 1995), where ganglioside in nervous tissue is composed of sialic acid as glycosphingolipids that are available in cerebral cortex of human brain at high concentration. Intriguingly, sialic acid extract and standard were found to be able to increase the number of active mitochondria significantly in SH-SY5Y cells compared with the control. With SH-SY5Y cells originally derived from a metastatic bone tumor biopsy, the number of active mitochondria in them seem to be able to effectively change when sialic acid was added to the media. Although there are many types of cell lines available to represent AD study *in vitro*, SH-SY5Y cell line is more pronounced if the study is focusing on mitochondrial dysfunction. Apart from its suitability for AD, it can also be applicable for other studies focused on mitochondrial dysfunction in other diseases.

## Conclusion

Our finding has indicated that MtMP measurement could be used to study mitochondrial dysfunction by *in vitro* technique*.* The increase observed in mitochondrial membrane potential in entire cell lines when subjected to sialic acid treatment indicated presence of healthy cells. This is vital to study on MtMP measurement for its effect on mitochondrial dysfunction. The sialic acid uptake also was noticed to occur with varying degrees in cell lines. Based on our findings among all the tested cells, SH-SY5Y was found to be the most suitable cell line especially for studies that will be focused on the expression of active mitochondria.

## Data Availability

The raw data supporting the conclusions of this article will be made available by the authors, without undue reservation.
